# Role of the tachykinin NK_1 _receptor in a murine model of cigarette smoke-induced pulmonary inflammation

**DOI:** 10.1186/1465-9921-10-37

**Published:** 2009-05-15

**Authors:** Katelijne O De Swert, Ken R Bracke, Tine Demoor, Guy G Brusselle, Guy F Joos

**Affiliations:** 1 Laboratory for Translational Research in Obstructive Pulmonary Diseases, Department of Respiratory Medicine, Ghent University Hospital, Ghent, Belgium

## Abstract

**Background:**

The tachykinins, substance P and neurokinin A, present in sensory nerves and inflammatory cells such as macrophages and dendritic cells, are considered as pro-inflammatory agents. Inflammation of the airways and lung parenchyma plays a major role in the pathogenesis of chronic obstructive pulmonary disease (COPD) and increased tachykinin levels are recovered from the airways of COPD patients. The aim of our study was to clarify the involvement of the tachykinin NK_1 _receptor, the preferential receptor for substance P, in cigarette smoke (CS)-induced pulmonary inflammation and emphysema in a mouse model of COPD.

**Methods:**

Tachykinin NK_1 _receptor knockout (NK_1_-R^-/-^) mice and their wild type controls (all in a mixed 129/sv-C57BL/6 background) were subjected to sub acute (4 weeks) or chronic (24 weeks) exposure to air or CS. 24 hours after the last exposure, pulmonary inflammation and development of emphysema were evaluated.

**Results:**

Sub acute and chronic exposure to CS resulted in a substantial accumulation of inflammatory cells in the airways of both WT and NK_1_-R^-/- ^mice. However, the CS-induced increase in macrophages and dendritic cells was significantly impaired in NK_1_-R^-/- ^mice, compared to WT controls, and correlated with an attenuated release of MIP-3α/CCL20 and TGF-β1. Chronic exposure to CS resulted in development of pulmonary emphysema in WT mice. NK_1_-R^-/- ^mice showed already enlarged airspaces upon air-exposure. Upon CS-exposure, the NK_1_-R^-/- ^mice did not develop additional destruction of the lung parenchyma. Moreover, an impaired production of MMP-12 by alveolar macrophages upon CS-exposure was observed in these KO mice. In a pharmacological validation experiment using the NK_1 _receptor antagonist RP 67580, we confirmed the protective effect of absence of the NK_1 _receptor on CS-induced pulmonary inflammation.

**Conclusion:**

These data suggest that the tachykinin NK_1 _receptor is involved in the accumulation of macrophages and dendritic cells in the airways upon CS-exposure and in the development of smoking-induced emphysema. As both inflammation of the airways and parenchymal destruction are important characteristics of COPD, these findings may have implications in the future treatment of this devastating disease.

## Background

Chronic obstructive pulmonary disease (COPD) is the fourth leading cause of death worldwide and a major burden on healthcare systems. Moreover, its prevalence and mortality are expected to escalate in the coming decades [1]. COPD is a chronic respiratory disease that is characterized by an abnormal inflammatory response of the lungs to noxious particles and gases. This leads to obstruction of the small airways and destruction of the lung parenchyma (emphysema), resulting in a slowly progressive development of airflow limitation that is not fully reversible [2,3].

Cigarette smoke is the major risk factor for the development of COPD, and it has been shown that smoking leads to airway inflammation with an increase of inflammatory cells of both the innate and adaptive immune system. Indeed, an exaggerated accumulation of macrophages [4,5], neutrophils [6,7], dendritic cells [8,9] and CD8^+ ^T-lymphocytes [10] has been observed in lungs of COPD patients. Moreover, in patients with severe COPD, lymphoid follicles containing T- and B-lymphocytes are present in the bronchial wall [11].

The tachykinins, substance P and neurokinin A, are present in sensory afferent nerves and inflammatory cells in the airways. They may be released by a variety of stimuli (e.g. cigarette smoke, ozone) and have various effects including smooth muscle contraction, facilitation of cholinergic neurotransmission, submucosal gland secretion, vasodilatation, increase in vascular permeability, stimulation of mast cells, B and T lymphocytes and macrophages, chemoattraction of eosinophils and neutrophils and the vascular adhesion of neutrophils [12].

Tachykinins mediate their effects by stimulation of tachykinin NK_1_, NK_2 _and NK_3 _receptors [13]. NK_1 _receptors are mainly involved in neurogenic inflammation (microvascular leakage and mucus secretion) while NK_2 _receptors are considered to be important in smooth muscle contraction. NK_3 _receptors have also been detected in the airways, and may have an important role in localized neural regulation of airflow to the lungs [14].

Several lines of evidence indicate a role for tachykinins in chronic obstructive pulmonary disease (COPD). Elevated levels of tachykinins have been recovered from the airways of patients with COPD [15]. Cigarette smoke, the main causative agent of COPD activates C-fiber endings, causing the release of tachykinins [16,17] and lowers the threshold for activation of these nerve endings [18]. Moreover, human alveolar macrophages possess functional NK_1 _receptors on their surface, which are upregulated in smokers [19]. In guinea pigs, chronic exposure to cigarette smoke increases the synthesis of substance P in jugular ganglia innervating the lung and airways [20]. Activation of C-fibers and the subsequent release of tachykinins induces neurogenic inflammation in the airways [21]. Furthermore, cigarette smoke-induced airway neutrophilia was attenuated by a dual tachykinin NK_1_/NK_2 _receptor antagonist in guinea pigs [22].

The purpose of this study was to characterize the precise role of the tachykinin NK_1 _receptor in a mouse model of cigarette smoke-induced COPD [23,24], more particularly in pulmonary inflammation, lymphoid follicle formation and development of pulmonary emphysema.

## Methods

### Animals

Tachykinin NK_1 _receptor knockout (NK_1_-R^-/-^) and wild type (WT) mice were derived as described from the mating of heterozygous tachykinin NK_1 _receptor mice [25]. The targeting construct was derived from a mouse 129/sv strain genomic library and targeted clones were injected into C57BL/6 blastocysts. Chimaeric males were mated with C57BL/6 females. The mice were bred from successive generations of sibling NK_1_-R^-/- ^and WT mice and can be thought of as representing a recombinant inbred strain. The NK_1_-R^-/- ^and WT breeding pairs were provided by the lab of S. Hunt (Cambridge, UK). The animals were bred locally and maintained in a conventional animal house in the animal research facilities of the Faculty of Medicine and Health Sciences, Ghent University Hospital and received food and water *ad libitum*. The NK_1_-R^-/- ^and WT mice were in good health and were fertile. No remarkable differences were observed between both genotypes. Male C57BL/6 mice were purchased from Harlan (Zeist, the Netherlands). The local Ethics Committee for animal experimentation of the faculty of Medicine and Health Sciences (Ghent, Belgium) approved all *in vivo *manipulations.

### NK_1 _receptor antagonist treatment

In a pharmacological validation experiment of sub acute CS-exposure C57BL/6 mice were treated daily – 30 minutes before air- or CS-exposure – with the NK_1 _receptor antagonist RP 67580 ((3a*R*,7a*R*)-Octahydro-2- [1-imino-2-(2-methoxyphenyl)ethyl]-7, 7-diphenyl-4*H*-isoindol) (Tocris, Bristol, UK) for 2 weeks. The antagonist was dissolved in 200 μl diluent (PBS with 20% DMSO) at a concentration of 0.1 μg/μl or 1 μg/μl and administered intraperitoneally. Control groups received IP injections of 200 μl diluent (PBS with 20% DMSO).

### Smoke exposure

Mice (male, 8–12 weeks, N = 8 per experimental group) were exposed whole body to the tobacco smoke of 5 cigarettes (Reference Cigarette 1R3, University of Kentucky, Lexington, KY) three times a day with 2 hours smoke-free intervals, 5 days a week for 4 (sub acute exposure) or 24 weeks (chronic exposure). An optimal smoke:air ratio of 1:12 was obtained. For the experiment with the NK_1 _receptor antagonist (RP 67580) mice (male, 8 weeks, N = 8 per experimental group) where exposed whole body to the tobacco smoke of 5 cigarettes (Reference Cigarette 3R4F without filter, University of Kentucky, Lexington, KY) four times a day with 30 minutes smoke-free intervals, 5 days a week for 2 weeks. An optimal smoke:air ratio of 1:6 was obtained. Smoke was generated with a standard smoking apparatus with the chamber adapted for groups of mice (chamber dimensions: 24 × 14 × 14 cm = 4700 cm^3^). The control groups were exposed to air. Carboxyhemoglobin in serum of smoke-exposed mice reached a non-toxic level of 8.3 ± 1.4% (compared to 1.0 ± 0.2% in air-exposed mice (n = 7 for both groups)), which is similar to carboxyhemoglobin blood concentrations of human smokers.

### Bronchoalveolar lavage

24 hours after the last smoke exposure, mice were killed with an overdose of pentobarbital (Sanofi, Libourne, France) and a tracheal cannula was inserted. 1 ml of Hank's balanced salt solution (HBSS), free of ionised calcium and magnesium but supplemented with 0.05 mM sodium EDTA was instilled 4 times via the tracheal cannula and recovered by gentle manual aspiration. The recovered bronchoalveolar lavage fluid (BALF) was centrifuged (1800 rpm for 10 min at 4°C). The supernatant was discarded and the cell pellet was washed twice and finally resuspended in 1 ml of HBSS. A total cell count was performed in a Bürker chamber and the differential cell counts on at least 400 cells were performed on cytocentrifuged preparations (Cytospin 2; Shandon Ltd., Runcorn, UK) using standard morphologic criteria after staining with May-Grünwald-Giemsa. Flow cytometric analysis of BAL-cells was also performed to enumerate dendritic cells.

### Lung digests

Immediately after bronchoalveolar lavage, the lung and systemic circulation was rinsed with saline supplemented with 5 mM EDTA. The left lung was used for histology, the right lung for the preparation of a cell suspension as detailed previously [23,24,26]. Briefly, the lung was thoroughly minced, digested, subjected to red blood cell lysis, passed through a 50 μm cell strainer, and kept on ice until labeling. Cell counting was performed with a Z2 Beckman-Coulter particle counter (Beckman-Coulter, Ghent, Belgium).

### Labeling of BAL-cells and lung single-cell suspensions for flow cytometry

Cells were pre-incubated with F_c_-receptor blocking antibody (anti CD16/CD32, clone 2.4G2) to reduce non-specific binding. Monoclonal antibodies used to identify mouse dendritic cell (DC) populations were: biotinylated anti-CD11c (N418 hybridoma, gift from M. Moser, Brussels Free University, Belgium) and phycoerythrin (PE)-conjugated anti-IA^b ^(AF6-120.1), followed by streptavidine-allophycocyanine (Sav-APC). We discriminated between macrophages and DCs using the methodology described by Vermaelen et al. [27]. After gating on the CD11c-bright population, two peaks of autofluorescence can be distinguished. Macrophages are identified as the CD11c-bright, high autofluorescent population, and do not express MHCII. DCs are identified as CD11c-bright, low autofluorescent cells, which strongly express MHCII. DCs enumerated by these criteria correspond with myeloid DCs. Mouse T-cell populations were characterized with the following monoclonal antibodies: fluorescein isothiocyanate (FITC)-conjugated anti-CD4 (L3T4), FITC-conjugated anti-CD8 (Ly-2) and biotinylated anti-CD3 (145-2C11). PE-conjugated anti-CD69 (H1.2F3) was used to evaluate the activation status of the T-cells. Biotinylated anti-CD3 was revealed by incubation with Sav-APC. All antibodies were obtained from Pharmingen (Beckton Dickinson, Erembodegem, Belgium). Finally, cell suspensions were incubated with 7-amino-actinomycin (7-AAD) to exclude dead cells (7-AAD positive cells). All labelling reactions were performed on ice with FACS-buffer. Flow cytometric data acquisition was performed on a dual-laser FACS Vantage™ flow cytometer running CELLQuest™ software (Beckton Dickinson, Erembodegem, Belgium). FlowJo software (Tree Star Inc. Ashland, OR) was used for data analysis.

### Histology

The left lung was fixated by intratracheal infusion of fixative (4% paraformaldehyde), as previously described [23,24,26]. After excision, the lung was immersed in fresh fixative during 2 h. The lung lobe was embedded in paraffin and cut in 3 μm transversal sections. Lung tissue samples were stained with hematoxylin and eosin, and examined by light microscopy for histological sections. For each animal, 10 fields at a magnification of 200× were captured randomly from the 4 different zones of the left lung (upper, middle upper, middle basal and basal zone) using a Zeiss KS400 image analyzer platform (KS400, Zeiss, Oberkochen, Germany).

### Quantification of emphysema

Emphysema is a structural disorder characterized by damage to the lung parenchyma. The destruction of the alveolar walls will lead to enlargement of the alveolar airspaces. The alveolar airspace enlargement was determined by mean linear intercept (L_m_) as described previously [23,28], using image analysis software (Image J 1.33). Only sections without cutting artefacts, compression or hilar structures (airway or blood vessel with a diameter larger than 50 μm) were used in the analysis. The L_m _was measured by placing a 100 × 100 μm grid over each field. The total length of each line of the grid divided by the number of alveolar intercepts gives the average distance between alveolated surfaces, or the L_m_. The Lm was measured by 2 independent observers, with a positive correlation (p < 0.01).

The destruction of alveolar walls was quantified by the DI [29]. A grid with 42 points that were at the center of hairline crosses was superimposed on the lung field. Structures lying under these points were classified as normal (N) or destroyed (D) alveolar and/or duct spaces. Points falling over other structures, such as duct walls, alveolar walls, etc. did not enter into the calculations. The DI was calculated from the formula: DI = D/(D + N) × 100.

### Morphometric quantification of lymphoid follicles

To evaluate the presence of lymphoid follicles in lung tissue after 24 weeks of smoke exposure, lung sections obtained from formalin-fixed, paraffin-embedded lung lobes were subjected to the following immunohistological CD3/B220 double staining [26,30]: at first, sections were incubated with Boehringer blocking reagent with triton and primary antibody anti-CD3, followed by goat-anti-rabbit biotin (both obtained from DakoCytomation). Then, slides were incubated with streptavidin horseradish peroxidase and colored with DAB. In a second step, sections were stained with anti-B220-biotin after Boehringer blocking (with triton). Finally, slides were incubated with streptavidin alkaline phosphatase (DakoCytomation) and colored with Vector blue (Vector Laboratories, Inc., Burlingame, California, USA). Lymphoid follicles were defined as accumulations of at least 50 cells and counted in the tissue area surrounding the airways (airway perimeter < 2000 μm). Results were expressed as counts relative to the numbers of airways per lung section.

### Immunohistochemistry for MMP-12

Sections obtained from formalin-fixed, paraffin-embedded lung lobes were subjected to the following immunohistological staining sequences [24]: blocking reagent, goat-anti-mouse MMP-12 (Santa Cruz Biotechnology, Santa Cruz, USA) or goat IgG isotype control and detection with Vectastain Elite Goat IgG ABC Kit (Vector, Burlingame, USA) and DAB substrate (DAKO, Glostrup, Denmark). Sections were counterstained with haematoxylin. The MMP-12 staining was simultaneously evaluated by two observers unaware of the treatment of the animals. The intensity of the MMP-12 staining was scored on a four point scale 0) none or very weak staining; 1) weak staining; 2) moderate staining; 3) intense staining.

### Measurement of chemokines

Using commercially available ELISA kits (R&D Systems), MIP-3α (Macrophage Inflammatory Protein-3α), KC (mouse IL-8) and activated TGF-β1 protein levels were determined in BAL fluid after 24 weeks of CS-exposure.

### Statistical analysis

All results are reported as mean ± standard error of the mean (SEM). Statistical analysis was performed with Sigma Stat software (SPSS 11.0 Inc, Chicago, IL, USA) using non-parametric tests (Kruskall-Wallis, Mann-Whitney U). P-values < 0.05 were considered as significant.

## Results

### CS-induced increase of inflammatory cells in BAL fluid and lung tissue

**Figure 1:**
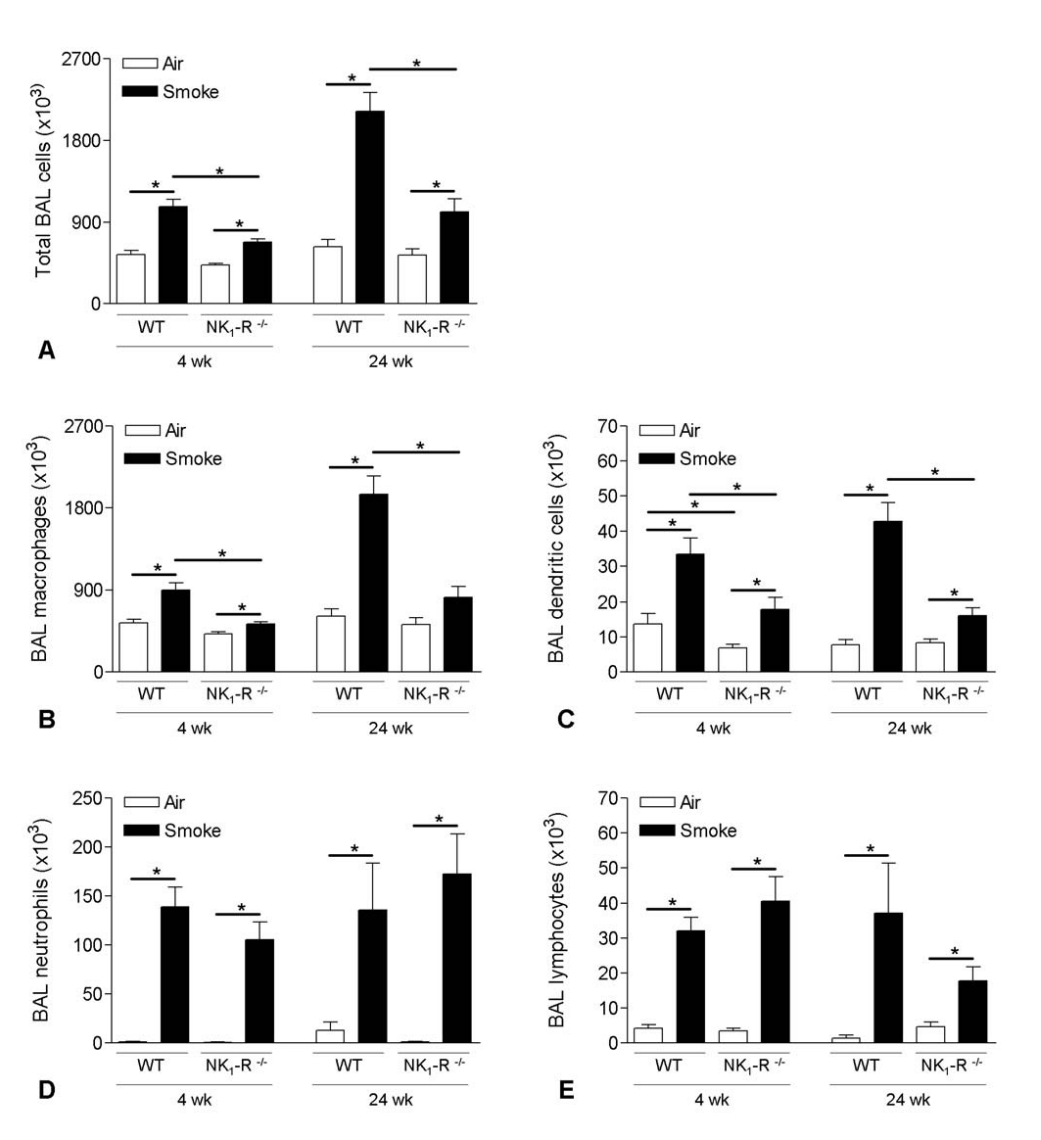
**Effect of cigarette smoke exposure on cell differentiation in bronchoalveolar lavage fluid**. Total bronchoalveolar lavage (BAL) cells and cell differentiation in BAL fluid of wild type and NK_1_-R^-/- ^mice upon sub acute (4 weeks) and chronic (24 weeks) exposure to air or cigarette smoke: **(A) **Total BAL cells, **(B) **macrophages, **(C) **dendritic cells, **(D) **neutrophils and **(E) **lymphocytes. Results are expressed as means ± SEM. N = 8 animals per group (* p < 0.05).

Both sub acute and chronic CS-exposure induced an enhanced accumulation of inflammatory cells in the bronchoalveolar lavage fluid, compared to air-exposed controls (Figure 1). Increased numbers of macrophages, DCs, neutrophils and lymphocytes were recovered by bronchoalveolar lavage in both CS-exposed tachykinin NK_1 _receptor WT and NK_1_-R^-/- ^mice (Figure 1). However, the CS-induced increase in total cells, macrophages and DCs was significantly attenuated in the NK_1_-R^-/- ^mice at both the sub acute and chronic time-point (Figure 1A–C). In contrast, no differences in the accumulation of neutrophils and lymphocytes were observed between smoke-exposed WT and NK_1_-R^-/- ^animals (Figure 1D–E). At the sub acute time-point, air-exposed NK_1_-R^-/- ^mice had significantly less DCs in their airways than WT control animals. This difference disappeared however with ageing in the chronic exposed group (Figure 1C).

In lung digests, sub acute and chronic CS-exposure induced increases in DCs and activated (CD69^+^) CD4^+ ^and CD8^+ ^T-lymphocytes. No differences were observed between WT and NK_1_-R^-/- ^animals (data not shown).

### Chronic CS-induced increase of peribronchial lymphoid follicles

**Figure 2:**
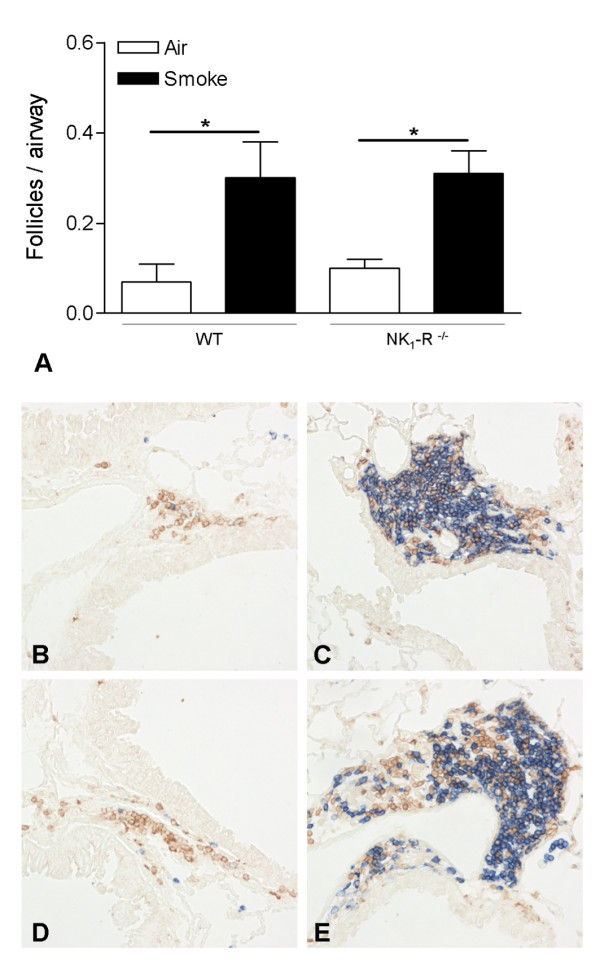
**Quantification of pulmonary lymphoid follicles upon chronic cigarette smoke exposure**. Peribronchial lymphoid follicles in lung tissue of wild type and NK_1_-R^-/- ^mice upon chronic (24 weeks) exposure to air or cigarette smoke (CS) **(A)**. Results are expressed as means ± SEM. N = 8 animals per group (* p < 0.05). Photomicrographs of peribronchial lymphoid follicles in lung tissue of air- and CS-exposed wild type and NK_1_-R^-/- ^mice at 24 weeks (chronic exposure; magnification ×200): **(B) **air-exposed wild type mice, **(C) **CS-exposed wild type mice, **(D) **air-exposed NK_1_-R^-/- ^mice and **(E) **CS-exposed NK_1_-R^-/- ^mice.

Immunohistochemistry using anti-CD3 and anti-B220 monoclonal antibodies, staining T- and B-lymphocytes respectively, revealed the presence of only a few small lymphoid follicles in lung tissue surrounding the airways of air-exposed WT and NK_1_-R^-/- ^mice (Figure 2). Chronic CS-exposure significantly increased the number of these peribronchal lymphoid follicles (Figure 2). There were no differences in follicle numbers between WT and NK_1_-R^-/- ^mice (Figure 2).

### Chronic CS-induced increase of inflammatory mediators in BAL fluid

To gain more insight into the mechanisms of airway inflammation in WT and NK_1_-R^-/- ^mice, we measured protein levels of MIP-3α/CCL20, KC (mouse homolog for IL-8) and activated TGF-β1 in BAL fluid supernatant.

**Figure 3:**
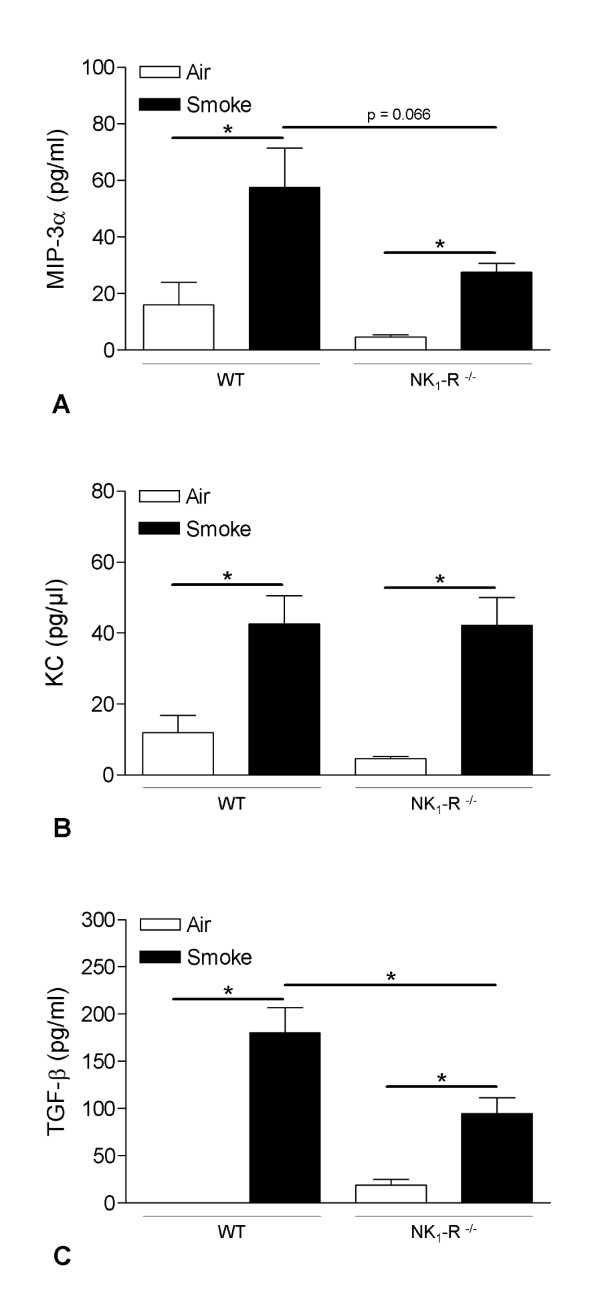
**Effect of chronic cigarette smoke exposure on the protein levels of inflammatory mediators in bronchoalveolar lavage fluid**. Protein levels of inflammatory mediators in the bronchoalveolar lavage fluid of wild type and NK_1_-R^-/- ^mice upon chronic (24 weeks) exposure to air or cigarette smoke, as measured by ELISA: **(A) **MIP-3α, **(B) **KC and **(C) **TGF-β1. Results are expressed as pg/ml (mean ± SEM). N = 8 animals per group (* p < 0.05). (MIP-3α: Macrophage Inflammatory Protein-3α; KC: mouse interleukin-8; TGF-β1: Transforming Growth Factor-β1).

Chronic CS-exposure significantly increased the levels of MIP-3α/CCL20 in both WT and NK_1_-R^-/- ^mice, compared to air-exposed controls. The increase in MIP-3α/CCL20 CS-exposed NK_1_-R^-/- ^mice was attenuated, compared to the CS-exposed WT mice, but this difference did not reach statistical significance (p = 0.066) (Figure 3A). Upon chronic CS-exposure, the protein levels of KC were equally increased in both WT and NK_1_-R^-/- ^mice (Figure 3B). Chronic CS-exposure significantly increased TGF-β1 concentrations in both genotypes, however the CS-induced increase in NK_1_-R^-/- ^mice was significanly impaired, compared to WT mice (Figure 3C).

### Chronic CS-induced development of pulmonary emphysema

**Figure 4:**
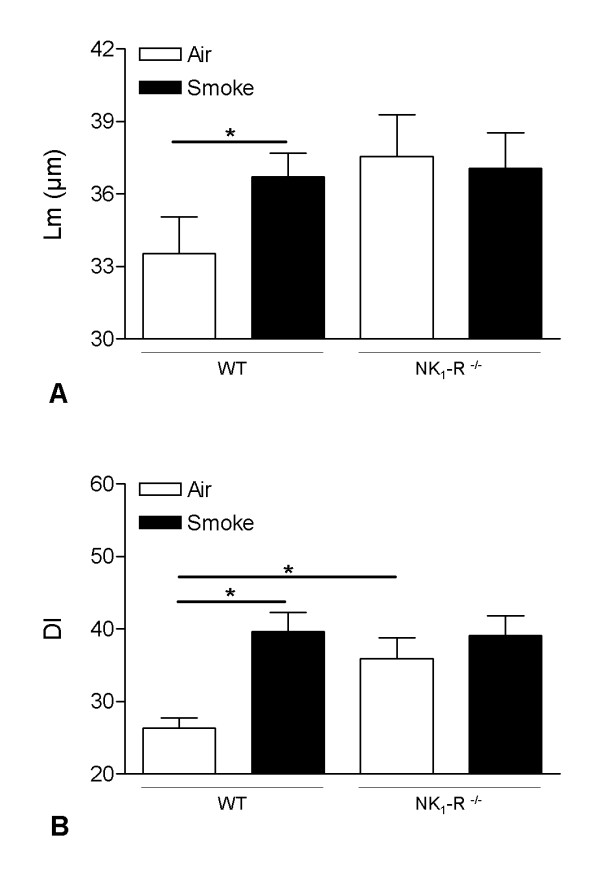
**Pulmonary emphysema upon chronic cigarette smoke exposure**. Mean linear intercept (Lm) **(A) **and destructive index (DI) **(B) **values of wild type and NK_1_-R^-/- ^mice upon chronic (24 weeks) exposure to air or cigarette smoke. Results are expressed as means ± SEM. N = 8 animals per group (* p < 0.05).

Evaluation of lung morphology demonstrated the presence of pulmonary emphysema in WT mice upon chronic CS-exposure, defined by an increased mean linear intercept (Lm) and destructive index (DI), compared to the air-exposed counterparts (Figure 4). No CS-induced increase in Lm or DI could be detected in NK_1_-R^-/- ^mice (Figure 4). However, baseline values of both Lm and DI were already higher in air-exposed NK_1_-R^-/- ^mice, compared to air-exposed WT mice.

### Chronic CS-induced increase of MMP-12 in lung macrophages

**Figure 5:**
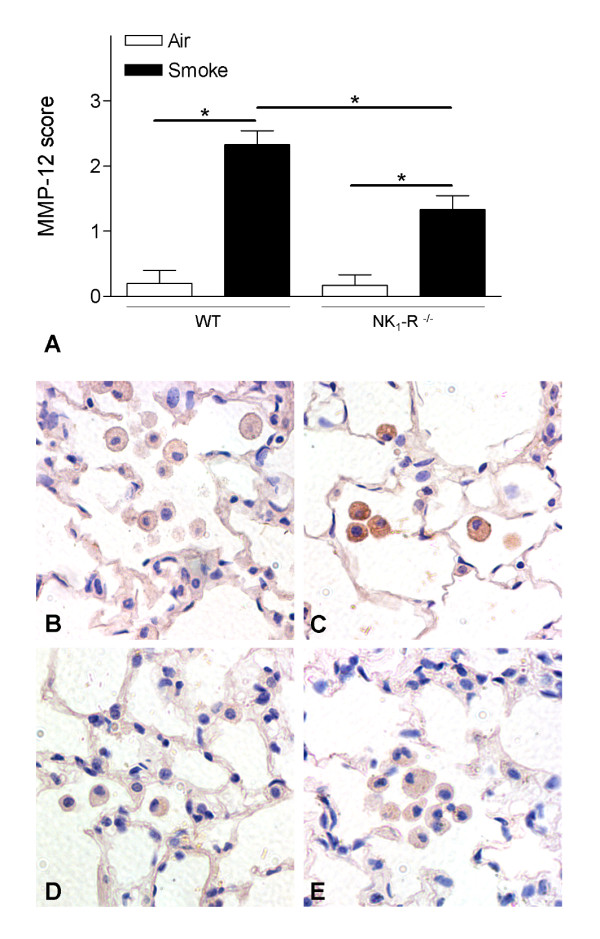
**Effect of chronic cigarette smoke exposure on the protein levels of MMP-12 in lung tissue**. Semiquantitative scoring of MMP-12 on immunohistochemical stained lung tissue sections of wild type and NK_1_-R^-/- ^mice upon chronic (24 weeks) exposure to air or cigarette smoke **(A)**. Photomicrograhps of immunohistochemistry for MMP-12 protein on lung tissue of wild type and NK_1_-R^-/- ^mice upon chronic (24 weeks) exposure to air or cigarette smoke (magnification ×400). **(B) **air-exposed wild type mice, **(C) **cigarette smoke-exposed wild type mice, **(D) **air-exposed NK_1_-R^-/- ^mice and **(E) **cigarette smoke-exposed NK_1_-R^-/- ^mice. Photomicrographs are representative of 8 animals per group.

Because MMP-12 is one of the major proteinases impicated in the development of pulmonary emphysema [31], we studied the presence of MMP-12 in lung tissue by immunohistochemistry. Chronic CS-exposure revealed significantly increased MMP-12 staining in macrophages of WT mice, compared to air-exposed controls (Figure 5). Interestingly, the MMP-12 induction upon CS-exposure was significantly attenuated in NK_1_-R^-/- ^mice, compared to WT mice (Figure 5).

### Effect of the NK1 receptor antagonist on CS-induced inflammation in BAL fluid

**Figure 6:**
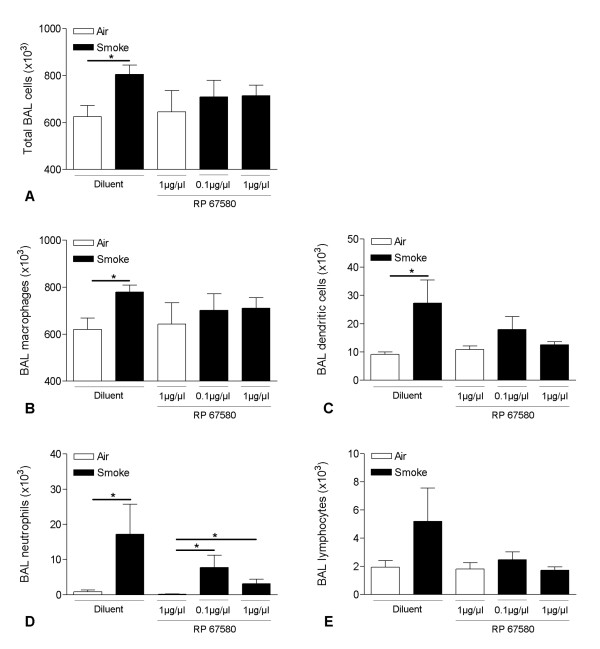
**Effect of the NK_1 _receptor antagonist on cigarette smoke-induced inflammation in bronchoalveolar lavage fluid**. Total bronchoalveolar lavage (BAL) cells and cell differentiation in BAL fluid of C57BL/6 mice upon IP injection with either 0.1 or 1 μg/μl of the NK_1 _receptor antagonist RP 67580 or diluent and subsequent exposure to air or cigarette smoke for 2 weeks: **(A) **Total BAL cells, **(B) **macrophages, **(C) **dendritic cells, **(D) **neutrophils and **(E) **lymphocytes. Results are expressed as means ± SEM. N = 8 animals per group (* p < 0.05).

Two weeks of CS-exposure significantly increased the numbers of total BAL cells, macrophages, dendritic cells and neutrophils in BAL fluid of C57BL/6 mice treated with diluent (Figure 6A–D). After daily IP injection with the NK_1 _receptor antagonist RP 67580, CS-exposure no longer induced a significant increase in the numbers of inflammatory cells in the BAL fluid, except for a significant increase in neutrophils (Figure 6).

## Discussion

In this mouse model of COPD, CS-exposure resulted in an increase of inflammatory cells in the lavage fluid whereby a role for the tachykinin NK_1 _receptor in macrophage and DCs accumulation was demonstrated. The impaired accumulation of these cell types seems, at least partially, mediated by the attenuated release of the chemokines MIP-3α/CCL20 and TGF-β1. Absence of the NK_1 _receptor already resulted in alveolar destruction in air-exposed mice. This alveolar enlargement did however not increase further upon chronic CS-exposure, which correlates with an impaired production of MMP-12 by alveolar macrophages in NK_1_-R^-/- ^mice. In a pharmacological validation experiment using a NK_1 _receptor antagonist (RP67580), we confirmed the protective effect of absence of the NK_1 _receptor on sub acute CS-induced pulmonary inflammation.

Macrophages and DCs are originally derived from monocyte precursors in the bone marrow [32,33]. During inflammation, increased amounts are recruited into the airway lumen and the alveoli. This can be mediated by either increased influx of precursors from the circulation or increased local proliferation or a combination of both. In vitro studies revealed a direct chemotactic activity of substance P through the NK_1 _receptor. Macrophages and DCs are known to express the functional tachykinin NK_1 _receptor [19,34,35] and are chemotactic towards substance P [36-39]. However, very high concentrations of agonist are needed for this phenomenon. In vivo, the half life of substance P is short as the peptide is quickly degraded by neutral endopeptidase. This peptidase is however inactivated by cigarette smoke [40] which can lead to increased levels of substance P in smoking animals and a direct chemotactic activity towards macrophages and DCs. Nevertheless, an indirect effect may be more likely as tachykinins can stimulate macrophages, epithelium, endothelium, mast cells and T cells to release mediators responsible for chemotaxis and transmigration of inflammatory cells through the vessel walls. We found an increased release of MIP-3α/CCL20 and TGF-β1 into the BAL fluid after CS-exposure, that was attenuated in the absence of the tachykinin NK_1 _receptor. The interaction of MIP-3α/CCL20 with its receptor CCR6 has been described as one of the most potent mechanisms for recruitment of immature DCs [41,42]. Moreover, we recently demonstrated an accumulation of immature Langerin^+ ^dendritic cells in the small airways of patients with COPD, which was associated with significantly increased expression of MIP3α/CCL20 in lungs and induced sputum of patients with COPD compared with "healthy" smokers without airway obstruction [43]. TGF-β1 has been shown to mediate recruitment of macrophages in COPD [44] and can also induce the differentiation of peripheral blood monocytes into DCs [45]. The lower levels of both MIP-3α/CCL20 and TGF-β1 in NK_1_-R^-/- ^mice can, at least partially, explain the reduced numbers of DCs and macrophages in these mice. Importantly, we confirmed the *in vivo *role of the NK_1 _receptor in CS-induced recruitment of macrophages and DCs by using the NK_1 _receptor antagonist RP 67580. Indeed, daily treatment with the antagonist prevented the significant CS-induced increase in macrophages and DCs that was seen in control animals.

In steady-state situations, airway macrophages are predominantly maintained by cell proliferation and to a lesser extent from monocyte precursor influx [46], while the rapid turn-over [33] of DCs suggest a continuous influx of precursors from the circulation. This different maintenance mechanism may explain why the macrophage population in naïve animals is not affected by the absence of the tachykinin NK_1 _receptor, while DC population is decreased. The precise mechanism responsible for this steady state influx is not known although age and environmental air quality seem to be involved. In the scope of this observation it is important to notice that DC levels from 'old' NK_1_-R^-/- ^mice did no longer differ from WT mice.

Despite the evidence for a chemotactic effect of substance P on neutrophils [36], no differences in neutrophil influx between NK_1 _receptor WT and NK_1_-R^-/- ^mice were observed. This correlated with equal amounts of the neutrophil attractant KC (the mouse homolog for IL-8) in both genotypes, but is in contrast with the observations of Matsumoto and colleagues. They reported that acute cigarette smoke-exposure of guinea pigs induced airway neutrophilia, which was inhibited with a dual tachykinin NK_1_/NK_2 _receptor antagonist [22]. However, the effect on airway neutrophilia in this study may be the result of blocking the NK_2 _receptor, which was left unblocked in the current study. Also, differences in duration of the smoke protocol and the resulting strenght of the inflammatory response may be of importance, as we did demonstrate an effect of the NK_1 _receptor antagonist RP 67580 on the neutrophil accumulation upon short time (2 weeks) CS-exposure.

Lymphocytes also express a functional tachykinin NK_1 _receptor [47] and are expected to migrate towards substance P [48]. However, the lack of the tachykinin NK_1 _receptor did not impair the CS-induced accumulation of lymphocytes in BAL fluid and lungs in our mouse model, nor did it affect the formation of peribronchial lymphoid follicles. These observations are in line with our previous work, where we demonstrated that the tachykinin NK_1 _receptor is not required for antigen-induced inflammatory cell influxes in the airway lumen of mice [49].

Chronic exposure to CS resulted in the development of pulmonary emphysema in WT mice. However, this enlargment of alveolar spaces was not observed in NK_1_-R^-/- ^mice. Alveolar destruction in pulmonary emphysema is believed to originate mainly from an imbalance between proteases and their inhibitors. Macrophages and DCs are the main sources of MMP-12, a matrix metalloproteinase that has been described as the key proteolytic enzyme in the development of CS-induced emphysema in mice [31]. The lower numbers of both macrophages and DCs in CS-exposed NK_1_-R^-/- ^mice should thus result in a diminished release of MMP-12 in these mice. Moreover, immunohistochemical staining showed impaired production of MMP-12 in alveolar macrophages of CS-exposed NK_1_-R^-/- ^mice. This correlates with the findings of Xu and colleagues, who described a significant correlation between substance P and MMP-12 in CS-exposed mice [50,51]. Other mechanisms that can lead to destruction of lung tissue, like alveolar cell apoptosis [52], should also be considered. Interestingly, Lucatelli and colleagues demonstrated a role for the NK_1 _receptor in lung epithelial cell death [53]. The possible protection against emphysema in the NK_1_-R^-/- ^mice should nevertheless be regarded with caution, as the air-exposed NK_1_-R^-/- ^mice already have enlarged alveolar spaces and more alveolar destruction, compared to the WT mice, which makes it difficult to compare CS-induced emphysema between WT and NK_1_-R^-/- ^mice. Baseline differences in lung morphology have already been described in other strains, such as C3H/HeJ mice [54].

As a therapeutic approach, blocking only the NK_1 _receptor is most likely insufficient, as most of the effects of tachykinins in the airways are mediated by more than one tachykinin receptor. Indeed, not only the NK_1_, but also NK_2 _and NK_3 _receptors can elicit features like airway smooth muscle contraction, vascular engorgement, mucus secretion, cholinergic nerve activation and recruitment of inflammatory cells [55,56]. Triple NK receptor antagonists have already been successful in reducing bronchoconstriction in patients with asthma [57], and could thus be ideal candidates for therapeutic intervention in COPD patients.

To conclude, the tachykinin NK_1 _receptor is involved in the accumulation of inflammatory cells in the airways during the inflammatory response to CS in a mouse model of COPD. As inflammation of the airways is an important characteristic of COPD, these findings may have implications in the future treatment of this devastating disease. Lower numbers of macrophages and DCs, combined with impaired release of MMP-12, also resulted in an attenuation of CS-induced pulmonary emphysema in NK_1_-R^-/- ^mice. However, further research is needed to unravel the precise mechanism by which signalling through the tachykinin NK_1 _receptor causes the increased accumulation of macrophages and DCs into the airway lumen upon cigarette smoke exposure and to clearly demonstrate a possible beneficial effect of tachykinin receptor antagonists in people suffering from COPD.

## Abbreviations

BAL: bronchoalveolar lavage; COPD: chronic obstructive pulmonary disease; CS: cigarette smoke; DC: dendritic cell; DI: destructive index; IL: Interleukin; L_m_: mean linear intercept; MIP-3α: Macrophage Inflammatory Protein-3α (CCL20); MMP-12: matrix metalloproteinase-12; TGF-β1: Transforming Growth Factor-β1.

## Competing interests

The authors declare that they have no competing interests.

## Authors' contributions

KDS carried out the design and coordination of the study, gathered the data on BAL and lung inflammation, interpreted the data and drafted the manuscript. KB quantified the inflammatory mediators, lymphoid follicles and MMP-12 IHC, carried out the pharmacological experiment, performed the statistical analysis, interpreted the data and drafted the manuscript. TDM performed the quantification of emphysema. GB participated in the coordination of the study, helped to interpret the data and critically revised the manuscript. GJ participated in the design and coordination of the study, helped to interpret the data and drafted the manuscript. All authors read and approved the final manuscript.
